# Editorial: From Oxidative Stress to Cognitive Decline - Towards Novel Therapeutic Approaches

**DOI:** 10.3389/fnmol.2021.650498

**Published:** 2021-04-12

**Authors:** Touqeer Ahmed, Nady Braidy

**Affiliations:** ^1^Neurobiology Laboratory, Department of Healthcare Biotechnology, Atta-ur-Rahman School of Applied Biosciences, National University of Sciences and Technology, Islamabad, Pakistan; ^2^Faculty of Medicine, Centre for Healthy Ageing, School of Psychiatry, University of New South Wales, Sydney, NSW, Australia

**Keywords:** oxidative stress, cognition, Alzheimer's disease, neurodegeneration, natural products

Oxidative stress is likely to play a major contributing role, or at least a causal role, in the etiology and pathogenesis of neurological disorders. So far, limited therapeutic options are available for people with these debilitating disorders. Oxidative stress is caused by multiple factors, including aging, greater vulnerability of easily oxidizable unsaturated fatty acids in the brain, higher metabolic activity by the brain, increased production of mitochondrial-derived free radicals, calcium dyshomeostasis, and glutamate-induced excitotoxicity. Moreover, environmental chemicals/toxins, heavy metals, and an imbalanced diet can contribute significantly to the accumulation of oxidative stress, potentially leading to reduced cellular viability, cognitive decline, and eventually death. In this special topic, a series of articles review recent trends in the molecular mechanisms of oxidative stress in the development and progression of neurodegenerative disorders, and highlight previously undescribed potential therapeutic options.

Oxidative stress represents a major factor which promotes neuronal degeneration, resulting in a wide variety of disorders, including mild cognitive impairment (MCI), which usually progresses to dementia. Arslan et al. systematically reviewed studies conducted on humans where several oxidative stress markers have been used as biomarkers in Alzheimer's disease (AD) at an early stage. The study found that non-invasive oxidative stress markers may be useful for the detection of MCI and AD, but are limited due to poor specificity and sensitivity. However, many of these markers may be used in combination with family history and other biochemical tests to detect the disease at an early stage. The study further showed that long-term use of selected vitamins and polyphenol-rich foods may be useful for managing AD pathology, although further work is needed to justify the use of natural antioxidants in clinic. Kandlur et al. reviewed the implications of age-associated oxidative damage on learning and memory and the molecular events, with special emphasis on associated epigenetic machinery. Increased understanding of these mechanisms may provide renewed insight in to the development of potential therapeutic targets within the oxidative system.

The cross-sectional study by Iqbal et al. further showed that, with increasing age, accumulation of metals was significantly associated with the tau and amyloid pathology. In addition, serum levels of various lipoproteins were associated with severity of MCI in a well-sized Pakistani population. Findings from this study can help in the translation of some commonly measured substances in serum levels to help in predicting the MCI. Hongo et al. explored carotenoid astaxanthin, mitigated memory deficits, and significantly reversed pathological hallmarks of AD, including Aβ42 deposition, pTau formation, GSH decline, and PV-positive neuronal deficits. In line with the clinical findings by Iqbal et al. elevated GSH levels may ameliorate pathological processes in the AD.

AD is characterized by progressive loss of basal forebrain cholinergic neurons and reduced cortical choline acetyltransferase activity is associated with cognitive decline. Most of the drugs available for the management of symptoms for AD are cholinergic modulators, and targeting the cholinergic system may be beneficial in neurocognitive disorders. The original research article by Kowalczyk et al., showed that Bergapten (furanocoumarin), a naturally occurring compound found in the Apiaceae family, could enhance memory performance against scopolamine-induced memory impairment. More specifically, the study demonstrated that Bergapten (both the single and repeated doses) increased memory acquisition and consolidation, suggesting its beneficial effects on multiple cognitive domains. Clinical translation of Bergapten is a promising approach to attenuate oxidative damage due to aging and age-related disorders.

Stroke is the leading cause of morbidity and mortality worldwide and represents a major risk factor for dementia. More than 85% of the stroke cases are ischemic, which result in major loss in physiological activity of the brain, leading to complex pathophysiological events culminating in cognitive decline and death. Ali et al. explored the neuroprotective effects of atorvastatin, cephalexin, and mycophenolate against NF-κB in ischemic stroke, as compared to the standard NF-κB inhibitor caffeic acid phenethyl ester (CAPE). The study demonstrated that these novels NF-κB inhibitors could attenuate ischemic stroke-induced neuronal toxicity by targeting NF-κB, a potential therapeutic approach in ischemic stroke, suggesting a frontier for emerging research. Furthermore, Ling et al. used a mouse model of middle cerebral artery occlusion to show that treatment with melatonin prior to ischemic injury could protect against neuroinflammation and oxidative stress and modulate the endogenously produced antioxidant enzyme, thioredoxin (Trx) pathway. These findings suggest that melatonin can represent a pharmacophore for the development of therapeutic strategies for enhancing recovery, prevention, and progression of ischemic injury.

Subarachnoid hemorrhage (SAH) represents another cerebrovascular disease with significant detrimental effects on the brain and cognitive performance. SAH can lead to endothelial cell injury and blood-brain barrier (BBB) disruption. The original research article by Zhuang et al. demonstrated that hydrogen inhalation could protect against SAH-induced endothelial cell injury, BBB disruption, microthrombosis, and vasospasm in rats, and neurobehavioral effects at least in part by inhibition of activation of the ROS/NLRP3 axis. Liu H. et al. demonstrated that Bakuchiol, an analog of resveratrol, could protect against early brain injury following SAH by ameliorating BBB disruption, oxidative stress, and apoptosis via modulation of Trx1/TXNIP expression and the phosphorylation of AMPK. Taken together, hydrogen inhalation and naturally occurring compounds such as Bakuchiol represent promising therapeutic strategies for protection against brain injury after SAH.

In recent years, there has been a concerted effort to identify and develop potential drugs with multi-target and multi-pathway neuroprotective effects in the treatment of neurocognitive disorders. Abdul Hannan et al. reviewed recent literature on the neuroprotective effects of phytochemicals that can co-activate cellular antioxidant defense and TrkB signaling-mediated cell survival systems. Yang et al. demonstrated that Catalpol (CAT), an iridoid glycoside compound, showed neuroprotective effects by attenuating microglial-mediated neuroinflammatory response through the inhibition of the p53-mediated Bcl-2/Bax/caspase-3 apoptosis pathway and regulating Keap1/Nrf2 pathway. These results collectively indicate the potential of CAT as a highly effective therapeutic agent for neuroinflammatory and neuro-oxidative disorders. In the original research article by Zhang et al., the authors used a cell culture system to demonstrate that the polyphenol Fisetin can protect against hyperglycemia-induced neurotoxicity through the PI3K/Akt/CREB pathway. Fisetin, which is abundant in fruits such as strawberries, grape seed, apple, and onion, can cross the BBB and demonstrated limited adverse effects. Liu P. et al. demonstrated that harmine, an analogous β-carboline alkaloid compound, ameliorated cognitive impairment by inhibiting NLRP3 inflammasome activation and enhancing the BDNF/TrkB signaling pathway in diabetic rats, and represents a potential therapeutic drug for diabetes-induced cognitive dysfunction. Dong et al. demonstrated that nicotine could protect against exposure hydrogen peroxide toxicity *in vitro* through the activation of the α7-nAChR/Erk1/2 signaling pathway, which suggests that nicotine usage may be a novel strategy for the treatment of neurodegenerative disorders associated with increased oxidative stress.

Apart from pharmacological therapeutic strategies for the treatment of neurocognitive disorders, non-pharmacological strategies have been developed to improve brain plasticity and minimize the neurotoxicity effects of amyloid-beta (Aβ) peptide. The original research article by Dare et al. used a neurotoxicity model induced by Aβ in rats to demonstrate that physical and cognitive exercise could reverse recognition memory deficits, lower hippocampal lipid peroxidation, and maintain optimal acetylcholinesterase activity following exposure to pathophysiological concentrations of Aβ. This suggests that physical and cognitive exercises may partially reverse hippocampal tissue disorganization.

Taken together, the articles add to our recent work in the area of oxidative stress and neurodegenerative diseases. Collectively, they demonstrate that oxidative stress plays a central role in the pathobiology of neurodegenerative diseases and AD in particular. The articles are suggestive of the potential beneficial effects of antioxidant therapy for the prevention and treatment of neurodegenerative diseases. These findings are mostly from preclinical studies; further support from clinical data can authenticate the use of these agents.

## Author Contributions

TA and NB wrote the manuscript and approved it. All authors contributed to the article and approved the submitted version.

## Conflict of Interest

The authors declare that the research was conducted in the absence of any commercial or financial relationships that could be construed as a potential conflict of interest.

